# Fluorescence Imaging in the Red and Far-Red Region during Growth of Sunflower Plantlets. Diagnosis of the Early Infection by the Parasite *Orobanche cumana*

**DOI:** 10.3389/fpls.2016.00884

**Published:** 2016-06-22

**Authors:** Carmen M. Ortiz-Bustos, María L. Pérez-Bueno, Matilde Barón, Leire Molinero-Ruiz

**Affiliations:** ^1^Department of Crop Protection, Institute for Sustainable Agriculture – Spanish National Research CouncilCordoba, Spain; ^2^Estación Experimental del Zaidín, Spanish National Research CouncilGranada, Spain

**Keywords:** broomrape, chlorophyll content, *Helianthus annuus* L., leaf pair, pre-symptomatic detection

## Abstract

Broomrape, caused by the root holoparasite *Orobanche cumana*, is the main biotic constraint to sunflower oil production worldwide. By the time broomrape emerges, most of the metabolic imbalance has been produced by *O. cumana* to sunflower plants. UV-induced multicolor fluorescence imaging (MCFI) provides information on the fluorescence emitted by chlorophyll (Chl) *a* of plants in the spectral bands with peaks near 680 nm (red, F680) and 740 nm (far-red, F740). In this work MCFI was extensively applied to sunflowers, either healthy or parasitized plants, for the first time. The distribution of red and far-red fluorescence was analyzed in healthy sunflower grown in pots under greenhouse conditions. Fluorescence patterns were analyzed across the leaf surface and throughout the plant by comparing the first four leaf pairs (LPs) between the second and fifth week of growth. Similar fluorescence patterns, with a delay of 3 or 4 days between them, were obtained for LPs of healthy sunflower, showing that red and far-red fluorescence varied with the developmental stage of the leaf. The use of F680 and F740 as indicators of sunflower infection by *O. cumana* during underground development stages of the parasite was also evaluated under similar experimental conditions. Early increases in F680 and F740 as well as decreases in F680/F740 were detected upon infection by *O. cumana.* Significant differences between inoculated and control plants depended on the LP that was considered at any time. Measurements of Chl contents and final total Chl content supported the results of MCFI, but they were less sensitive in differentiating healthy from inoculated plants. Sunflower infection was confirmed by the presence of broomrape nodules in the roots at the end of the experiment. The potential of MCFI in the red and far-red region for an early detection of *O. cumana* infection in sunflower was revealed. This technique might have a particular interest for early phenotyping in sunflower breeding programs. To our knowledge, this is the first work where the effect of a parasitic plant in its host is analyzed by means of fluorescence imaging in the red and far-red spectral regions.

## Introduction

*Orobanche cumana* Wallr. is a holoparasitic plant that parasitizes sunflower (*Helianthus annuus* L.) and limits crop yield in all the countries of Southern Europe and areas around the Black Sea where sunflowers are grown, as well as in North Africa, Israel, and China (Molinero-Ruiz et al., 2015). On average, sunflower seed losses caused by *O. cumana* (broomrape) can be quantified as being above 50% when susceptible hybrids are grown, and they frequently reach 100% in heavily infested fields (Domínguez, 1996; Jestin et al., 2014). Crop yield reductions due to infection by *O. cumana* depend on several factors, such as aggressiveness of the parasite, sunflower genotype, earliness of broomrape emergence, soil depth and, particularly, the soil infestation level or seed-bank in the soil (Molinero-Ruiz et al., 2009; Jestin et al., 2014). Germination of *O. cumana* seeds in the soil is stimulated by chemical compounds exuded in the rhizosphere by surrounding sunflower roots (Joel et al., 2011; Yoneyama et al., 2011; Raupp and Spring, 2013; Ueno et al., 2014). The seed produces a radicle that grows toward the host root, reaches it, and adheres to the host plant. *Orobanche* spp. obtain water and inorganic compounds from the host through a xylem to xylem contact, and, unlike the vast majority of parasitic plants, they also utilize the carbon fixed by the host plants, which is transferred through phloem continuity between host and parasite (Molinero-Ruiz et al., 2015). Penetration of the intrusive cells into the xylem triggers their division, leading to the formation of a tubercle, a callus-shaped bulge of parasite cells outside the root (Molinero-Ruiz et al., 2015). In *O. cumana*-sunflower interaction, the utilization of host photoassimilates by the parasite results in depletion of resources which are necessary for the growth of sunflower and for the optimal development of the seeds.

The complete disruption of the host vascular system of sunflower forced by the parasitism of *O. cumana*, begins during the initial weeks of the crop. In fact, and as early as 3 weeks after inoculation, a decrease in secondary metabolites is observed in the host upon infection by the parasite (Pérez-Bueno et al., 2014). After underground development of tubercles parasite stems emerge aboveground at around flowering-time of sunflower plants (Melero-Vara and Alonso, 1988). By that time most of the metabolic imbalance has been produced irreversibly by the parasite to sunflower. From that moment onward, the impact of *O. cumana* on the crop yield is evident through absent or small sized capitula, their low number and small seeds, and even plant death (Molinero-Ruiz et al., 2015). Because the parasite establishes itself in the host plant in underground stages, non-destructive methods for diagnosing that establishment are greatly needed. Additionally, diagnosis tools used as early as possible after plant sowing and parasite infection will have a wide range of applications in the search for efficient control approaches, such as genetic resistance or treatments with potential biocontrol agents.

Multicolor fluorescence imaging (MCFI) is a technique based on induction of fluorescence emission through UV excitation. It provides leaf images of the autofluorescence emitted by plants in four spectral bands peaking at around 440 nm (blue, F440), 520 nm (green, F520), 690 nm (red, F690), and 740 nm (far-red, F740; Buschmann and Lichtenthaler, 1998; Buschmann et al., 2000). The first two fluorescence signals (F440 and F520) are related to secondary metabolites (Harris and Hartley, 1976; Morales et al., 1996) while the other two (F680 and F740) are emitted by Chl *a* (Buschmann et al., 2000). Also of particular interest are the fluorescence ratios; the red/far-red (F680/F740) ratio was found to be a good indicator of stress-induced decreases in Chl content (Hák et al., 1990; Buschmann, 2007). Therefore, MCFI is considered to be a powerful tool for the research on plant physiology related to development (Lang et al., 1994; Cerovic et al., 1999; Subhash et al., 1999). Moreover, this imaging technique is especially valuable for detecting early symptoms in leaves as those caused by *Tobacco mosaic virus* in tobacco plants (Chaerle et al., 2007) and *Pseudomonas syringae* in bean plants (Pérez-Bueno et al., 2015).

On the other hand, the use of Chl meters for quantifying leaf Chl levels has become common in recent years. Unlike traditional leaf Chl content estimation techniques consisting mainly of pigments extraction by organic solvents followed by *in vitro* measurements with a spectrophotometer, Chl content meters allow to asses this leaf parameter in a fast and non-destructive way. Furthermore, previous studies using Chl meters have shown that they provide good estimates of leaf Chl content (Fanizza et al., 1991; Brito et al., 2011). Other useful applications of Chl meters are the estimation of foliar N concentration (Chang and Robison, 2003; Liu et al., 2012) and crop disease detection (Calderón et al., 2014).

The objectives of this study were to: (a) analyze the distribution of red and far-red fluorescence emission during early growth stages of healthy sunflower plants, (b) evaluate the use of fluorescence imaging in the red and far-red region as an indicator of sunflower infection by *O. cumana* during underground development stages of the parasite, and (c) compare fluorescence imaging in the red and far-red region with measurements of total leaf Chl content as indicator of early infection by *O. cumana.*

## Materials and Methods

### Distribution of Red and Far-Red Fluorescence Emission in Healthy Sunflower Plantlets

#### Healthy Plant Material

Four sunflower seeds of the inbred line NR5 were surface-disinfected by soaking them in 20% household bleach (50 g of active chlorine per liter) for 5 min, then seeds were rinsed in deionised water for 5 min and kept in a dark incubator at 25°C at saturation humidity until radicles were 2–5 mm long. After germination, seeds were individually transplanted into small pots with 250 g of a soil mixture (sand:silt:peat moss 2:1:1, *V*). Plants were grown in a glasshouse at 12–22°C without additional lighting for 5 weeks and watered as needed. When plants were 2-weeks-old and once a week for the next 2 weeks of growth, they were fertilized with 15 ml/pot of a nutrient solution with N:P:K (7:5:6).

#### Multicolor Fluorescence Imaging

Multicolour fluorescence imaging was carried out using an Open FluorCamFC 800-O and analyzed using the Fluorcam7 software (Photon Systems Instruments, Brno, Czech Republic) according to Pérez-Bueno et al. (2015). Fluorescence images in red (F680) and far-red (F740) regions of the spectrum (which are emitted by chlorophyll *a*) were acquired sequentially after excitation with UV light (360 nm). Images corresponding to the F680/F740 ratio were calculated by the mentioned software. Fully developed leaf pairs (LPs) were measured from 2 weeks after transplanting. Thereafter, they were measured twice a week for 3 weeks until the end of the experiment. Measurements were always taken at the same time of day on attached and unshaded leaves.

The F680 and F740 emission by the leaf of healthy sunflower plants was studied by defining three different leaf areas (base, apex, and a region in the middle of the leaf). In addition, progress of chlorophyll fluorescence emission on the first four LPs of the plants was analyzed.

#### Statistical Analyses

The F680 and F740 emission in leaf areas of healthy sunflower was analyzed in a completely randomized design. Eight replicates were considered and one blade of an LP was regarded as a replicate. Significant differences in the intensity of F680 and F740 between leaf areas was calculated using analysis of variance (ANOVA). If significant effects were obtained, Fisher’s protected LSD tests (*P* = 0.05) were used as the comparison procedure. In the case of the time course of the F680 and F740 in LP, means and standard errors of 8–12 replicates were considered. Both experiments were repeated twice and ANOVA analysis was conducted to determine significant differences between the whole set of data. As no significant differences were found, data were pooled.

### Red and Far-Red Fluorescence Emission, and Leaf Chl Content in Sunflower Plantlets upon Inoculation with *O. cumana*

#### *Orobanche cumana* Inoculation and Sunflower Plant Growth

Five sunflower seeds (replications) of the inbred line NR5, which is the differential for race F (Molinero-Ruiz et al., 2015), were grown in the greenhouse for 5 weeks under the same conditions as those previously described and used as controls. In the case of inoculated plants, five NR5 seedlings were individually transplanted into a soil mixture uniformly infested with 10 mg of *O. cumana* seeds of LPA13 population previously characterized as race F (García-Carneros et al., 2014). From then on, the experiment was carried out under the same conditions described in the section on healthy sunflower plantlets.

At the end of the experiment, sunflower plants were uprooted and their roots carefully cleaned to remove soil, washed and air-dried. Nodules of *O. cumana* in each plant were counted using a stereo microscope and weighed with a precision balance.

#### Measurements of the Total Leaf Chlorophyll Content

The total content on Chl was determined by an Chl content meter (CL-01 Hansatech Instruments, Ltd., Kings Lynn, UK), which provides a rapid and direct way to determine the relative content of Chl in leaves. Measurements were taken twice a week, from 2 and until 5 wai, in control and in inoculated plants. In each plant, one measurement (replicate) was made in the middle of all fully developed leaves, avoiding the main vein. For each time point, all LPs were individually analyzed. Between 12 and 20 replicates (measurements) were considered for each LP.

#### Spectrophotometric Chlorophyll Quantification

At the end of the experiment (5 wai), and before plants were uprooted, Chl *a* and Chl *b* content were determined spectrophotometrically for the second, third and fourth LP of four control and four inoculated plants. Two 1 cm diameter leaf disks were collected from each LP, weighted and immediately placed in vials and frozen in liquid nitrogen. Each sample was ground, the total pigments extracted with 4 ml of a solution of 80% acetone (v/v). The absorbance was measured at 470, 647 and 663 nm with a Shimadzu UV-1800 spectrophotometer (Shimadzu Corporation, Tokyo, Japan). Total Chl content and the Chl *a*/*b* ratio were calculated according to Lichtenthaler and Buschmann (2001).

#### Statistical Analyses

Sunflower plants were inoculated in two experiments which were set up as a factorial on a completely randomized design. Since ANOVA analysis did not detect any significant difference of data between them, the data were pooled. For each time point and LP, data of MCFI and Chl content included a maximum of 20 measurements. Sometimes, MCFI measurements were not taken on completely horizontal leaves resulting in a reduction to 14 replications for that particular time point. Similarly, if Chl content was determined on leaves that were too small to be clamped or had a very irregular surface, measurements were omitted and the replication number was reduced to 12. In any case, and for each time point, the same number of replications of control and inoculated plants was used in the statistical analyses. Significant differences in fluorescence parameters, broomrape incidence, total Chl content and Chl *a/b* ratio between inoculated and control plants were analyzed using the same statistical procedures as explained for the healthy sunflowers.

## Results

### MCFI Distribution Pattern on Healthy Sunflower Plants

**FIGURE 1:**
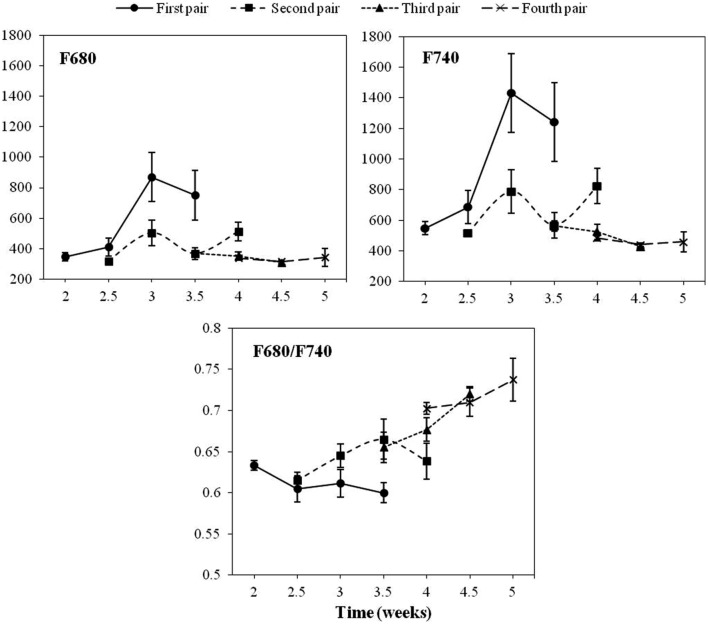
**Progress of chlorophyll fluorescence emission on the first, second, third and fourth pair of true leaves of a healthy sunflower plant.** Error bars represent the standard error of the mean of 8–12 replications. Time is expressed as weeks after transplanting.

**Figure 1** shows the average values of F680, F740 and their ratio for the first four LPs of healthy sunflower plants from the second to the fifth week of growth. Values of F680 and F740 of the first LP were significantly higher in the third week as compared to those recorded 1 week earlier. However, the F680/F740 ratio of the same LP showed a slight decrease in the third week. Concerning the second LP, no significant changes were detected in any of the fluorescence parameters analyzed. The third LP showed a significant increase in the F680/F740 ratio during the measurement period. Conversely, the other two variables decreased slightly throughout the same period. The three fluorescence parameters of the fourth LP remained constant during the measuring week (**Figure 1**).

The pattern of fluorescence across the leaves was found to be very homogeneous and therefore not dependent on measurement location on the leaf (data not shown). Consequently, a similar size area was selected in the middle of the leaf for the subsequent image analysis of healthy vs. *O. cumana* infected plants.

### Alterations in MCFI Distribution Pattern on Sunflower Plants upon Infection with *O. cumana*

**FIGURE 2:**
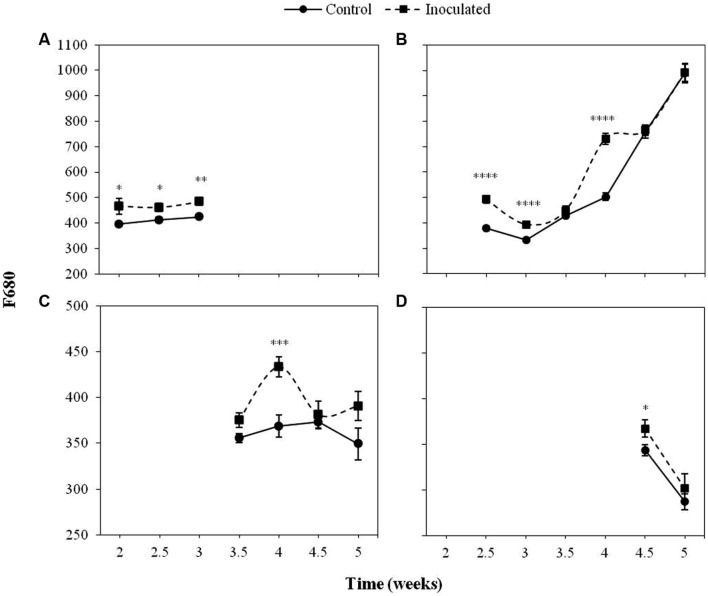
**Mean measurements of F680 of the first (A), second (B), third (C), and fourth (D) leaf pair (LP) of sunflower plants inoculated with *Orobanche cumana* and control plants.** Vertical bars represent the standard error of the mean of 14–20 replications. Analyses of variance were conducted and asterisks indicate significant differences (^∗^*P* < 0.05; ^∗∗^*P* < 0.01; ^∗∗∗^*P* < 0.001; ^∗∗∗∗^*P* < 0.0001).

**FIGURE 3:**
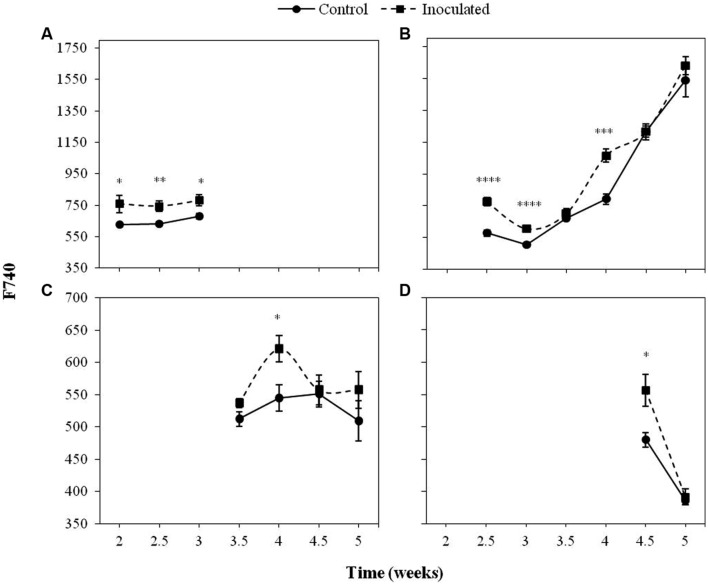
**Mean measurements of F740 of the first (A), second (B), third (C), and fourth (D) LP of sunflower plants inoculated with *O. cumana* and control plants.** Vertical bars represent the standard error of the mean of 14–20 replications. Analyses of variance were conducted and asterisks indicate significant differences (^∗^*P* < 0.05; ^∗∗^*P* < 0.01; ^∗∗∗^*P* < 0.001; ^∗∗∗∗^*P* < 0.0001).

**Figures 2** and **3** show the progress of F680 and F740, respectively in the first four LPs of sunflower plants inoculated with *O. cumana* compared with that of control plants.

The most informative parameters were those of the first LP from the second to the third week of growth and the second LP from the fourth week on. Between 2 and 3 wai, F680 and F740 of the first LP was significantly higher (*P <* 0.05) in the inoculated sunflowers as compared to the controls. In the same period of time, F680 and F740 signals in the second LP of inoculated plants were significantly higher than those of the controls (*P <* 0.05), as well as at 4 wai (*P <* 0.05). F680 and F740 signals of the third and fourth LP of inoculated plants were significantly higher than those of the controls at 4 wai in the third LP (*P <* 0.05) and half a week later in the fourth LP (*P <* 0.05).

**FIGURE 4:**
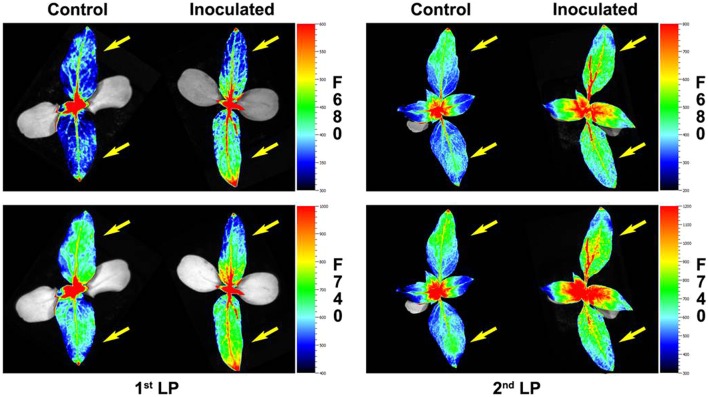
**Red fluorescence (F680) and far- red fluorescence (F740) images of the two first LP of sunflower plants inoculated with *O. cumana* and control plants at 2 and 2.5 growth weeks respectively.** First LPs are marked with an arrow.

Representative images of the two first LPs from control and inoculated sunflower are shown in **Figure 4**.

**FIGURE 5:**
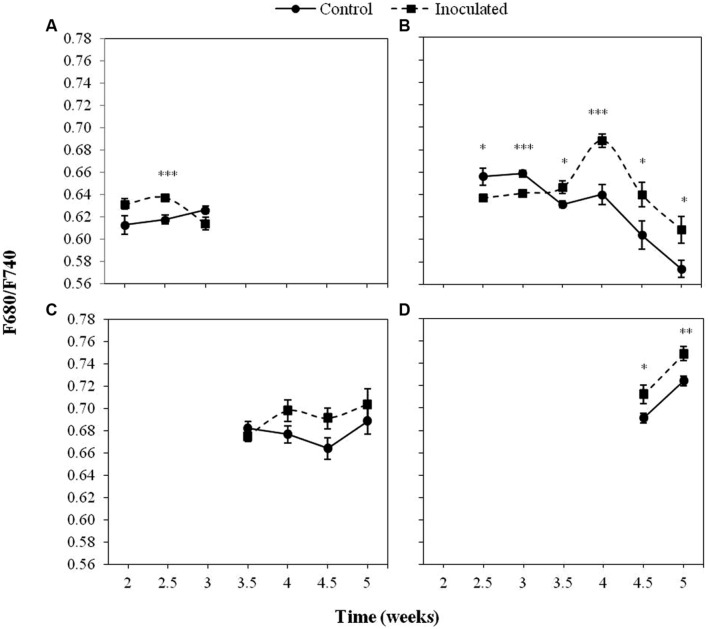
**Mean measurements of the F680/F740 ratio of the first (A), second (B), third (C), and fourth (D) LP of sunflower plants inoculated with *O. cumana* and control plants.** Vertical bars represent the standard error of the mean of 14–20 replications. Analyses of variance were conducted and asterisks indicate significant differences (^∗^*P* < 0.05; ^∗∗^*P* < 0.01; ^∗∗∗^*P* < 0.001).

**Figure 5** displays the values of the F680/F740 ratio in inoculated and control sunflower plants in the first four LPs as well as significant differences between treatments. A significant effect (*P <* 0.05) of the inoculation by *O. cumana* was observed in F680/F740 of the second LP, being lower in inoculated plants at 2.5 and 3 wai (*P <* 0.05), and higher during the rest of the measurements (*P <* 0.05). Significant differences (*P <* 0.05) of F680/F740 between treatments were obtained in the fourth LP (averages 0.73 and 0.71 for inoculated and control plants, respectively). Although no significant differences (*P* > 0.05) were obtained between inoculated and control plants when the F680/F740 ratio of the third LP was analyzed, similar trends were observed.

### Chlorophyll Content in Sunflower Plants upon Infection with *O. cumana*

**FIGURE 6:**
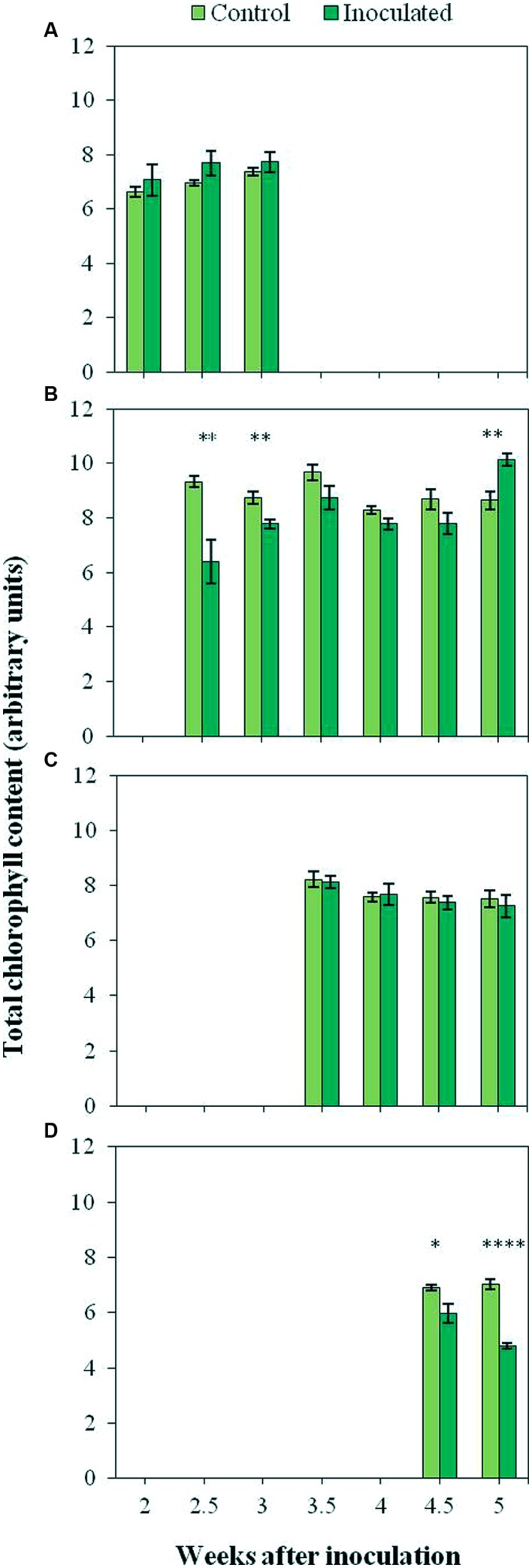
**Total chlorophyll content of the first (A), second (B), third (C), and fourth (D) LP of control plants and plants inoculated with *O. cumana.*** Vertical bars represent the mean of 12–20 independent measurements with their standard error. Analyses of variance were conducted at *P* < 0.05 and asterisks indicate significant differences (^∗^*P* < 0.05; ^∗∗^*P* < 0.01; ^∗∗∗∗^*P* < 0.0001).

The time course of the total Chl content of the first four LPs (A to D) is presented in **Figure 6**. Differences between the Chl content of inoculated and control plants depended on the LP analyzed, being significantly low (*P <* 0.05) in the second LP of inoculated plants at nearly all time points (7.70 arbitrary units as compared to 8.94 in the controls). The measurements conducted on the fourth LP resulted also in significantly (*P <* 0.05) lower values of inoculated plants as compared to the controls (5.38 and 6.98 arbitrary units respectively). In the case of the first and the third LP, no significant (*P >* 0.05) differences in total Chl content were detected between inoculated and control plants.

Spectrophotometric measurements of Chl content are presented in **Supplementary Figure S1**, which shows the differences in the total Chl content of each LP between control and inoculated plants. A significant (*P <* 0.05) increase in the total Chl content of the second LP (**Supplementary Figure S1**) was observed in inoculated plants as compared to the controls (1659.90 and 1460.19 μg/g fresh weight, respectively). On the contrary, measurements of the fourth LP resulted in significantly (*P <* 0.05) lower total Chl content in inoculated plants than in control plants (1050.90 and 1639.31 μg/g fresh weight, respectively). No significant (*P >* 0.05) differences of total Chl content were detected in the third LP. Neither differences in the Chl a/b ratio of LPs were observed between inoculated and control plants (data not shown).

### Determination of Final Degree of Sunflower Infection by *O. cumana*

When sunflower plants were inoculated with *O. cumana*, both inoculated and control plants developed four LPs at the end of the experiment (5 wai). No symptoms of parasite infection were observed in the inoculated plants throughout the experiment. Nevertheless, the presence of the parasitic plant in inoculated sunflowers was confirmed by the existence of tubercles in the roots at the end of the experiment. While no tubercles were observed in the controls, means of 24.4 tubercles/plant and 0.46 g tubercles/plant were obtained in inoculated sunflowers (data not shown).

## Discussion

During our study, the emission of red and far-red fluorescence in healthy sunflower depended on the LP analyzed. F680 and F740 nm showed the same trend in all the LPs analyzed, with higher F740 values compared to those of F680, probably due to the partial reabsorption of the red fluorescence emitted by leaf Chl *a* (Gitelson et al., 1998). Since F680/F740 was inversely correlated with the Chl content of leaves (Hák et al., 1990; Buschmann and Lichtenthaler, 1998), low values found in the first LP during early stages of the growth of sunflower, compared with the other three LPs under study, were indicative of higher Chl contents. A similar trend of low F680/F740 was observed in the case of the second LP, although with some delay in the time. In our study, the role of an LP as leader in Chl content was sequentially acquired by the one immediately above, and this happened approximately every 1–2 weeks. A different physiological activity between cotyledons and the first LP of sunflower, in terms of photosynthesis, has been reported by Jucknischke and Kutschera (1998). Also, differences in Chl content and photosynthesis have been reported as being dependent on leaf position in sunflower (Connor and Sadras, 1992; Subrahmanyam and Rathore, 2004). The correlation between photosynthetic activity and Chl content in individual LPs upon infection with *O. cumana* should be investigated in the future.

Increases in both F680 and F740 upon infection of *O. cumana* were observed in all LPs analyzed compared to that of the healthy controls in agreement with previous results by our group (Pérez-Bueno et al., 2014). The evolution of F680/F740 throughout the time was similar to that of F680 and F740 in the same LP. Values of F680/F740 increased for the second to fourth LPs upon infection by *O. cumana* and at different times in each LP, which could be indicative of a decrease in the Chl content upon infection assuming a similarity in the results reported by Gitelson et al. (1999), where the ratio F700/F730 was presented as an indicator of Chl content. Similarly, to those observed in F680 and F740 of LP from healthy sunflowers, the decreases were detected in different LPs sequentially in the time. In inoculated plants, higher values of F680/F740 in the first LP until the third week after inoculation indicated lower Chl contents. From the third week on, a decrease in Chl content as compared to that of the controls occurred in the second LP of the plants. The same trend was observed in the third and fourth LPs when measurements of inoculated and control plants were compared later on in the time. The alteration in F680 and F740 due to *O. cumana* infection shows the usefulness of the MCFI as an early indicator of parasite infection, as was suggested in a previous work (Pérez-Bueno et al., 2014). Furthermore, our results showed that the best indicators of parasite infection were the measurements of F680, F740, and F680/F740 in the first and second LPs from the second to the third week of plant growth. These findings confirm previous results by our research group about early detection of parasite infection by means of fluorescence emission of the first LP of sunflower. Even 3 wai proposed by Pérez-Bueno et al. (2014) as the earliest time for discriminating infected from uninfected plants, was reduced in this work to 2 wai. Therefore, the non-destructive pre-symptomatic disease diagnosis might be feasible as early as 15 days after sunflower inoculation.

Low Chl contents in LP from infected plants at certain times were also shown by outputs of the portable Chl-meter, which showed significantly low Chl contents in the second LP 4.5 weeks after inoculation and in the fourth LP from that moment on. Our results also reveal the greater sensitivity and accuracy of MCFI in diagnosing sunflower infection by *O. cumana* as compared to the direct measurements of Chl in intact leaves, because trends observed when using the hand-held Chl-meter were statistically significant according to red and far-red Chl fluorescence emissions. The positive correlation between outputs of portable Chl-meters and Chl fluorescence measurements has been reported by other authors (Netto et al., 2002, 2005; Nyi et al., 2012; Hu and Fang, 2013). In our work, F680 and F740 in parasitized plants were in agreement with direct measurements of Chl, particularly when the second and fourth LPs were considered. In addition, the spectrophotometric measurements of Chl content at 5 wai provided further evidence that, upon parasite infection, the Chl content in sunflower leaves decreased. The positive correlation between outputs of portable Chl-meters and photosynthetic pigments extracted from leaves has been reported by many authors (Fanizza et al., 1991; Netto et al., 2005; Brito et al., 2011). Finally, the greater sensitivity of MCFI compared to measurements of Chl content might be related to the fact that fluorescence intensity is not only dependent on the Chl content, but also on other factors such as leaf architecture or chloroplast organization (Cerovic et al., 1999). The effect of parasite infection on them might be explored in the future.

Early studies detected a decrease in Chl content in leaves of *Trifolium repens* upon infection by *O. minor*, although no effect on the host photosynthesis was observed (Dale and Press, 1998). Some authors, such as Hibberd et al. (1999) and Watling and Press (2001), suggested that the high competitiveness of holoparasites as a source forces an increase in host photosynthesis. On the contrary, other authors reported low rates of photosynthetic activity of the host upon infection by the holoparasite, such as in the case of tomato plants infected by the broomrape *O. ramosa* (Mauromicale et al., 2008) or *Mikania micrantha* infected by *Cuscuta campestris* (Shen et al., 2007). It is widely known that Chl fluorescence is an excellent approach to photosynthetic efficiency, which can directly or indirectly reflect the impact of biotic and abiotic stresses on plants. Hence, a number of studies have used chlorophyll fluorescence imaging to examine the impact of plant pathogens, i.e., virus, bacteria, and fungi, on host photosynthesis, as excellently reviewed by Rolfe and Scholes (2010). In future research, imaging measurements of Chl fluorescence kinetics in sunflower upon infection by *O. cumana* might explore on the plant photosynthesis alteration as a consequence of the holoparasite infection.

Generally, MCFI has been applied to the study of local symptoms in leaves caused by infections of airborne pathogens (Chaerle et al., 2007; Konanz et al., 2014; Pérez-Bueno et al., 2015). However, symptoms in leaves can as well be a consequence of an infection that occurred far beyond those organs, such as roots in the case of soilborne pathogens (Granum et al., 2015). In the latter, the responses observed in leaves clearly relate to plant defense mechanisms and the alteration of the host physiology due to the infection, since any direct damage in them can be discarded. Up to now, plant infections by soilborne pathogens have only been addressed by means of MCFI in Granum et al. (2015). Rousseau et al. (2015) have recently proved the potential of Chl fluorescence to detect the effect of *Phelipanche ramosa* on *Arabidopsis thaliana*. However, to the best of our knowledge, this is the first time that the impact of a root parasite on its plant host is examined by using fluorescence imaging in the red and far-red region. This inexpensive, non-destructive technic has proved to be an effective diagnostic tool in the early detection of sunflower plants parasitized by *O. cumana.* Moreover, the parameters F680 and F740 detect the parasite infection as early as 2 weeks after inoculation, and this might have a particular interest for early phenotyping in sunflower breeding programs.

## Author Contributions

LM-R, MP-B, and MB: conceived and designed the experiments. CO-B, LM-R, and MP-B: conducted experiments. CO-B and LM-R analyzed data and interpreted the results. MP-B: mounted images. LM-R and MB: contributed materials, equipment, and analysis tools. CO-B and LM-R wrote the manuscript and all the authors reviewed it and approved the final version.

## Conflict of Interest Statement

The authors declare that the research was conducted in the absence of any commercial or financial relationships that could be construed as a potential conflict of interest. The reviewer DM declared a past co-authorship with one of the authors LM-R to the handling Editor, who ensured that the process met the standards of a fair and objective review.

## Supplementary material

FIGURE S1 **Total chlorophyll content for the second, third and fourth LP of sunflower plants inoculated with *O. cumana* and control plants.** Vertical bars represent the average of 8 independent measurements with their standard error. Analyses of variance at *P* < 0.05 between inoculated and control plants were conducted and asterisks indicate significant differences (^∗^*P* < 0.05; ^∗∗∗^*P* < 0.001).
