# Supplementary figures and images for: Fluorescence Imaging in the Red and Far-Red Region during Growth of Sunflower Plantlets. Diagnosis of the Early Infection by the Parasite Orobanche cumana
Source: Front Plant Sci. 2016 Jun 22;7:884. doi: 10.3389/fpls.2016.00884 (PMC4916182; doi:10.3389/fpls.2016.00884)

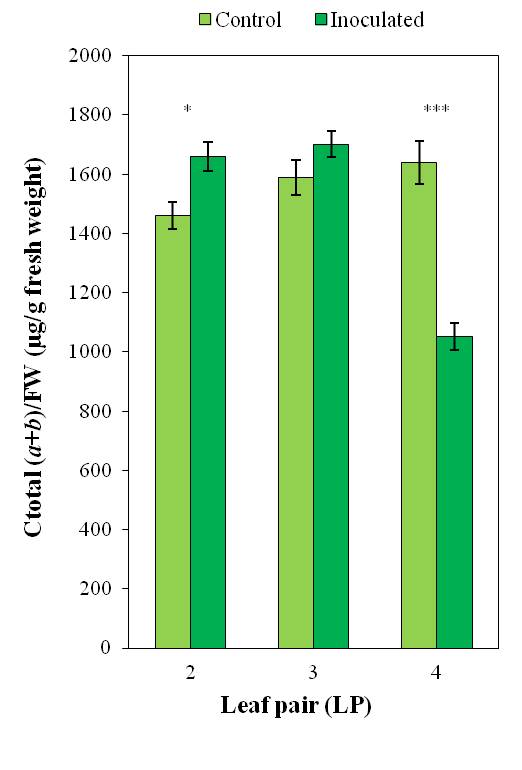

Supplement: FIGURE S1 — Total chlorophyll content for the second, third and fourth LP of sunflower plants inoculated with O. cumana and control plants. Vertical bars represent the average of 8 independent measurements with their standard error. Analyses of variance at P < 0.05 between inoculated and control plants were conducted and asterisks indicate significant differences (∗P < 0.05; ∗∗∗P < 0.001). [file Image_1.JPEG]
